# Dissection of Quorum-Sensing Genes in *Burkholderia glumae* Reveals Non-Canonical Regulation and the New Regulatory Gene *tofM* for Toxoflavin Production

**DOI:** 10.1371/journal.pone.0052150

**Published:** 2012-12-20

**Authors:** Ruoxi Chen, Inderjit K. Barphagha, Hari S. Karki, Jong Hyun Ham

**Affiliations:** Department of Plant Pathology and Crop Physiology, Louisiana State University Agricultural Center, Baton Rouge, Louisiana, United States of America; University of the West of England, United Kingdom

## Abstract

*Burkholderia glumae* causes bacterial panicle blight of rice and produces major virulence factors, including toxoflavin, under the control of the quorum-sensing (QS) system mediated by the *luxI* homolog, *tofI*, and the *luxR* homolog, *tofR*. In this study, a series of markerless deletion mutants of *B. glumae* for *tofI* and *tofR* were generated using the suicide vector system, pKKSacB, for comprehensive characterization of the QS system of this pathogen. Consistent with the previous studies by other research groups, *ΔtofI* and *ΔtofR* strains of *B. glumae* did not produce toxoflavin in Luria-Bertani (LB) broth. However, these mutants produced high levels of toxoflavin when grown in a highly dense bacterial inoculum (∼ 10^11^ CFU/ml) on solid media, including LB agar and King’s B (KB) agar media. The *ΔtofI*/*ΔtofR* strain of *B. glumae*, LSUPB201, also produced toxoflavin on LB agar medium. These results indicate the presence of previously unknown regulatory pathways for the production of toxoflavin that are independent of *tofI* and/or *tofR*. Notably, the conserved open reading frame (locus tag: bglu_2g14480) located in the intergenic region between *tofI* and *tofR* was found to be essential for the production of toxoflavin by *tofI* and *tofR* mutants on solid media. This novel regulatory factor of *B. glumae* was named *tofM* after its homolog, *rsaM*, which was recently identified as a novel negative regulatory gene for the QS system of another rice pathogenic bacterium, *Pseudomonas fuscovaginae*. The *ΔtofM* strain of *B. glumae*, LSUPB286, produced a less amount of toxoflavin and showed attenuated virulence when compared with its wild type parental strain, 336gr-1, suggesting that *tofM* plays a positive role in toxoflavin production and virulence. In addition, the observed growth defect of the *ΔtofI* strain, LSUPB145, was restored by 1 µM *N-*octanoyl homoserine lactone (C8-HSL).

## Introduction

*Burkholderia glumae*, the primary causal agent of bacterial panicle blight (BPB) of rice, is one of the most important disease problems affecting rice production in the southern United States, including Louisiana, Arkansas and Texas [1]. This rice disease has also been reported from many rice-growing areas around the world, including east Asia, southeast Asia and South America [1]. The optimal temperature range for the growth of *B. glumae* is 38–40°C, but this bacterium can also grow at temperatures as high as 50°C [2]. A typical characteristic of *B. glumae* is the production of the bright yellow phytotoxin, toxoflavin, which is a major virulence factor of this pathogen [3]–[6].

In *B. glumae*, production of major virulence factors, including toxoflavin, is dependent on the quorum-sensing (QS) system mediated by a pair of LuxI and LuxR homologs, TofI and TofR [4], [7], [8]. QS is a cell-to-cell communication mechanism that allows bacterial cells to collectively behave like a multicellular organism. In Gram-negative bacteria, QS systems mediated by LuxI and LuxR-family proteins are involved in a diverse range of bacterial behaviors and traits, including formation of biofilm, production of virulence factors, conjugation, and antibiosis [9], [10]. The LuxI/LuxR system, which is considered the prototype of the QS systems of Gram-negative bacteria, was first discovered from *Vibrio fischeri*, a luminous symbiont in marine animals [11], [12]. LuxI-family proteins are synthases that produce *N-*acyl homoserine lactone (AHL)-type intercellular signal molecules; LuxR-family proteins are cognate receptors that specifically bind to the AHL molecules [13].

Two types of AHL molecules, *N-*octanoyl homoserine lactone (C8-HSL) and *N-*hexanoyl homoserine lactone (C6-HSL), are synthesized by the LuxI-family protein of *B. glumae*, TofI [4]. It is thought that the LuxR-family protein of *B. glumae*, TofR, specifically binds to C8-HSL and the resultant TofR-C8-HSL complex triggers the production of toxoflavin by activating the transcription of *toxJ*, which has a *lux* box-like *cis* element (*tof-*box) upstream of the coding sequence for the binding of the TofR-C8-HSL complex [4]. Unlike C8-HSL, functions of C6-HSL in *B. glumae* and other *Burkholderia* spp. remain unknown. ToxJ encoded by *toxJ* is required for the transcription of *toxR*; and ToxR, a LysR-type transcriptional regulator, in turn activates the expression of the *toxABCDE* and *toxFGHI* operons, which harbor gene clusters for toxoflavin biosynthesis and transport, respectively [4]. This regulatory cascade (the TofI/TofR QS system → ToxJ → ToxR → *toxABCDE* and *toxFGHI*) is considered to be the central regulatory system for the production of toxoflavin, which may allow *B. glumae* to attack host cells in accordance with its population levels at infection sites [4]. Nevertheless, the genetic functions of *tofI* and *tofR* as well as additional components of the QS system governing the expression of bacterial virulence genes in *B. glumae* are not fully understood.

In this study, a series of deletion mutants deleted in the QS genes, *tofI* and *tofR*, were successfully generated from the U.S. virulent strain, 336gr-1 [2], for the further characterization of the QS system and the related global regulatory network in *B. glumae*. Through the genetic analyses conducted in this study, previously unknown *tofI-* and/or *tofR-*independent pathways for the production of toxoflavin were revealed and a new regulatory gene required for these pathways, *tofM*, was discovered between the *tofI* and *tofR* loci.

## Materials and Methods

### Bacterial Strains, Plasmids, Media and Growth Conditions

The bacterial strains and plasmids used in this study are listed in Table 1. All the *Escherichia coli* and *B. glumae* strains were routinely grown or maintained in LB broth or on LB agar plates [14] at 30°C or 37°C (even though the original definition of LB was corrected by Bertani as ‘lysogeny broth’ [15], the terms, ‘LB broth’ and ‘LB agar’, are used to clearly contrast two different growth conditions tested in this study). Bacterial strains grown in liquid media were incubated in a shaking incubator at 200 rpm. LB agar plates amended with 30% sucrose were used to counter-select the recombinant mutants that lost the sucrose-sensitive gene, *sacB*, through the secondary homologous recombination. The levels of bacterial growth and toxoflavin production in liquid or solid media were determined in four different growth conditions; LB alone, LB with 1 µΜ C6-HSL (Sigma-Aldrich, St. Louis, MO, USA); LB with 1 µΜ C8-HSL (Sigma-Aldrich, St. Louis, MO, USA); and LB with both 1 µΜ C6-HSL and 1 µΜ C8-HSL. The antibiotics and their working concentrations used in this study were: ampicillin (Amp), 100 µg/ml; kanamycin (Km), 50 µg/ml; nitrofurantoin (Nit), 100 µg/ml; gentamycin (Gm), 20 µg/ml; and tetracycline (Tc), 20 µg/ml.

**Table 1:** Bacterial strains and plasmids used in this study.

Strain or Plasmid	Description	Reference
*Escherichia coli*		
DH10B	F^−^ *araD139* Δ(*ara, leu*)7697 Δ*lacX74 galU galK rpsL deoR* ø80d*lacZ*ΔM15 *endA1 nupG recA1* *mcrA* Δ(*mrr hsdRMS mcrBC*)	[28]
DH5α	F^−^ *endA1 hsdR17* (r_k_^−^, m_k_^+^) *supE44 thi-1 λ* ^−^ *recA1 gyrA96 relA1 deoR* Δ (*lacZYA*-argF)-U169 ø80d*lacZ*ΔM15	[28]
S17-1λpir	*recA thi pro hsdR* [res- mod+][RP4::2-Tc::Mu-Km::Tn*7*] λ *pir* phage lysogen, Sm^r^/Tp^r^	[29]
*Burkholderia glumae*		
336gr-1	Wild type strain isolated from diseased rice in Crowley, Louisiana, USA	This study
LSUPB139	A Δ*tofI-tofR* derivative of 336gr-1	This study
LSUPB145	A Δ*tofI* derivative of 336gr-1	This study
LSUPB169	A Δ*tofR* derivative of 336gr-1	This study
LSUPB201	A Δ*tofI/*Δ*tofR* derivative of 336gr-1	This study
LSUPB286	A Δ*tofM* derivative of 336gr-1	This study
LSUPB292	A Δ*tofR/*Δ*tofM* derivative of 336gr-1	This study
LSUPB293	A Δ*tofI/*Δ*tofM/*Δ*tofR* derivative of 336gr-1	This study
LSUPB294	A Δ*tofI/*Δ*tofM* derivative of 336gr-1	This study
*Chromabacterium violaceum*		
*C. violaceum* CV026	A biosensor that produces a purple pigment in the presence of AHL molecules	[20]
Plasmid		
pBBR1MCS-2	A broad host range cloning vector, RK2 *ori*, *lacZα*, Km^R^	[30]
pBBR1MCS-5	A broad host range cloning vector, RK2 *ori*, *lacZα*, Gm^R^	[30]
pBBtofIM	A subclone of pBBtofIMR for the 2,808-bp *tofI/tofM* region inserted into pBBR1MCS-5 at the*Bgl*II and *Sac*I sites, Gm^R^	This study
pBBtofIMR	A subclone of pCos808 for the 3,670-bp *tofI/tofM/tofR* region inserted into pBBR1MCS-2 atthe *Eco*RI and *Sac*I sites, Km^R^	This study
pBBtofM	A *tofM* clone in pBBR1MCS-5, Gm^R^	This study
pBBtofRM	A subclone of pBBtofIMR for the 1,925-bp *tofR/tofM* region inserted intopBBR1MCS-2 at the *EcoR*I and *Pvu*II sites, Km^R^	This study
pCos808	The cosmid clone harbouring *tofI*, *tofM* and *tofR*, Amp^R^	This study
pJP5603	A suicide vector, R6K *γ-ori*, RP4 *oriT*, *lacZ*’, Km^R^	[31]
pJPtofIUD	A suclone of pLDtofIUD containing the upstream and downstream flanking regions of *tofI* in pGP5603, Km^R^	This study
pKKSacB	A suicide vector; R6K γ-*ori*, RP4 *oriT*, *sacB*, Km^R^	(Ham andBarphagha, *unpublished*)
pKKSacBΔtofI	A subclone of pJPtofIUD for the upstream and downstream flanking regions of *tofI* in pKKSacB, Km^R^	This study
pKKSacBΔtofIMR	A subclone of pLDtofIDRD carrying the downstream flanking regions of *tofI* and *tofR* in pKKSacB, Km^R^	This study
pKKSacBΔtofM	A plasmid carrying the upstream and downstream flanking regions of *tofM* in pKKSacB, Km^R^	This study
pKKSacBΔtofR	A subclone of pKKtofRUD for the upstream and downstream flanking regions of *tofR* in pKKSacB, Km^R^	This study
pKKSacBtofMU	A subclone of pSCtofMU for a upstream flanking region of *tofM* in pKKSacB, Km^R^	This study
pKKtofRD	A subclone of pSCtofRD for the downstream flanking region of *tofR* in pKNOCK-Km, Km^R^	This study
pKKtofRUD	A subclone of pSCtofRU for the upstream flanking region of *tofI* in pKKtofRD, Km^R^	This study
pKNOCK-Km	A suicide vector; R6K γ-*ori*, RP4 *oriT*, Km^R^	[32]
pLD55	A suicide vector; f1 *ori*, R6K *γ-ori*, RP4 *oriT*, *lacZα*, Amp^R^, Tc^R^	[19]
pLDtofID	A subslone of pSCtofID for the downstream flanking region of *tofI* in pLD55, Amp^R^, Tc^R^	This study
pLDtofIDRD	A subclone of pSCtofRD for the downstream flanking region of *tofR* in pLDtofID, Amp^R^, Tc^R^	This study
pLDtofIUD	A subclone of pSCtofIU for the upstream flanking region of *tofI* in pLDtofID, Amp^R^, Tc^R^	This study
pRK2013::Tn*7*	A helper plasmid; ColE1 *ori*	[16]
pSC-A-amp/kan	A blunt-end PCR cloning vector; f1 *ori*, pUC *ori*, *lacZ*’, Km^R^, Amp^R^	Stratagene
pSCtofID	A clone of the 512 *bp* downstream flanking region of *tofI* in pSC-A-amp/kan, Amp^R^, Km^R^	This study
pSCtofIU	A clone of the 545*-*bp upstream flanking region of *tofI* in pSC-A-amp/kan, Amp^R^, Km^R^	This study
pSCtofM	A clone containing the 986-bp region of *tofM* and its upstream region, Amp^R^, Km^R^	This study
pSCtofMD	A PCR clone of the 412-bp downstream flanking region of *tofM* in pSC-A-amp/kan, Amp^R^, Km^R^	This study
pSCtofMU	A PCR clone of the 433-bp upstream flanking region of *tofM* in pSC-A-amp/kan, Amp^R^, Km^R^	This study
pSCtofRD	A clone of the 829-bp downstream flanking region of *tofR* in pSC-A-amp/kan, Amp^R^, Km^R^	This study
pSCtofRU	A clone of the 426-bp upstream flanking region of *tofR* in pSC-A-amp/kan, Amp^R^, Km^R^	This study

### Recombinant DNA Techniques

Routine DNA cloning and amplification procedures were conducted following standard methods [14]. PCR products used for cloning were purified using the QuickClean 5 M PCR Purification Kit (GenScript, Piscataway, NJ, USA) and cloned into pSC-A-amp/kan using the Strata Clone™ PCR cloning kit (Agilent Technologies, Santa Clara, CA, USA). Genomic DNA of the wild type and mutant strains were extracted using the GenElute™ Bacterial Genomic DNA kit (Sigma-Aldrich, St. Louis, MO, USA). Electroporation for transforming *E. coli* cells was conducted with a GenePulser unit (BioRad Laboratories, Hercules, CA, USA) at 1.5 kV with 2 µl DNA and 25 µl competent cells. Triparental mating using the helper plasmid, pRK2013::Tn*7* [16], was used to transform *B. glumae* [17]. DNA were extracted from agarose gels using the GenElute™ Gel Extraction Kit (Sigma-Aldrich, St. Louis, MO, USA). DNA sequencing was performed by the LSU School of Veterinary Medicine Gene Lab. DNA concentrations were measured using a NanoDrop DN-1000 Spectrophotometer (Thermo Scientific, Wilmington, DE, USA). The genomic library of *B. glumae* 336gr-1 was created previously in our laboratory [18].

### Allelic Exchange of the *B. glumae* Genome for Targeted Deletions

A DNA construct in pKKSacB for deleting a target gene(s) was first introduced into a parental *B. glumae* strain via conjugation, following a previously described method [18]. Because *B. glumae* is resistant to Nit and pKKSacB contains a Km-resistance gene, the recombinant *B. glumae* strain in which a DNA construct in pKKSacB is integrated in the genome via single homologous recombination was initially selected on LB agar medium containing Km and Nit. Subsequently, the selected strain was grown overnight at 30°C in LB broth without any antibiotics. To select the mutants with secondary homologous recombination between the integrated DNA construct and the genome, which would result in the eviction of the integrated DNA construct and consequently the deletion of the target gene(s), the overnight culture was spread on LB agar medium containing 30% sucrose to select sucrose-resistant colonies. Individual sucrose-resistant colonies of *B. glumae* were then tested for the sensitivity to Km to screen marker-less deletion mutants. Deletion of target gene(s) in each of the selected Km-sensitive and sucrose-resistant mutants was confirmed by the appropriate diagnostic PCRs. Deletions of *tofI*, *tofR*, *tofM* were confirmed using the primer sets, TofI(H)F/TofI(H)R, TofR(H)F/TofR(H)R, and orf1-CT-F/orf1-CT-R, respectively, while the deletion of the entire *tofI-tofR* region in LSUPB139 was confirmed using the primer set, TofI(H)F/TofR(H)R. Primer sequences and PCR conditions for individual primer sets are described in Table 2.

**Table 2:** Primers and PCR conditions used in this study.

Primer name	Primers* (5′ → 3′)	Annealing and extension conditions
dtof1	ACTGGTACC TCGAACCCGACTCCG	Annealing: 60°C/30 sExtension: 72°C/1 min
dtof2	GGATCC AGCTCGGCGGCGATATGG	
dtofI3	GGATCC ACATCGACGCGCAGACGC	Annealing: 62°C/30 sExtension: 72°C/1 min
dtofI4	GCACTAGTATCCGCCCGAGATCCG	
TRD3	GGATCC GCGCGAACGCGAGGTGC	Annealing: 65°C/30 sExtension: 72°C/1 min
TRD6	ACTAGT ACGGCGTGACCGGCGTC	
TofR BF	AGGATCCGCTGCTCGTTTTCC	Annealing: 55°C/30 sExtension: 72°C/1 min
TofR BR	GACTAGTATCAGATTGCTGCG	
TofI(H)F	GTTCGTCAACGACGACTACG	Annealing: 53°C/30 secExtension: 72°C/2.5 min
TofR(H)R	CATGAGCATGGAAAAGAGCA	
TofI(H)F	GTTCGTCAACGACGACTACG	Annealing: 54°C/30 sExtension: 72°C/1 min
TofI(H)R	CGGAATTACCACGAGGACAC	
orf1-CT-F	ATGGTCAACAGTCCGAACACGC	Annealing: 58°C/30 sExtension: 72°C/1 min
orf1-CT-R	TCATGGGCTGCTTAAACGCAGAAG	
TofR(H)F	AAGAATGACAGCGTGGAAGC	Annealing: 50°C/30 sExtension: 72°C/1 min
TofR(H)R	CATGAGCATGGAAAAGAGCA	
tofI-jh1	GTCTACGTATTGGGACGCGAT	Annealing: 55°C/30 sExtension: 72°C/30 s
tofI-jh2	ACAGCCGCTCGATGCTGCAGA	
UPHP- FP	GGATCC ACATGCCGAAGTC	Annealing: 50°C/30 sExtension: 72°C/1 min
UPHP- RP	ACTAGT GTAGGGATGAAGCA	
DwN-FP	ACTAGT CGCTGGTCGCAC	Annealing: 50°C/30 sExtension: 72°C/1 min
DwN-RP	TCTAGA GAATTTTTCGTCTTC	

* Restriction sites (underlined) introduced in primers: GGTACC (*Kpn*I), GGATCC (*Bam*HI), ACTAGT (*Spe*I), and TCTAGA (*Xba*I). Default PCR conditions were: initial denaturation, 95°C/5 min; denaturation, 94°C/30 s; number of cycles, 30; and final extension, 72°C/7 min.

### DNA Constructs for the Targeted Deletions of *tofI*, *tofM*, and *tofR*

**Figure 1:**
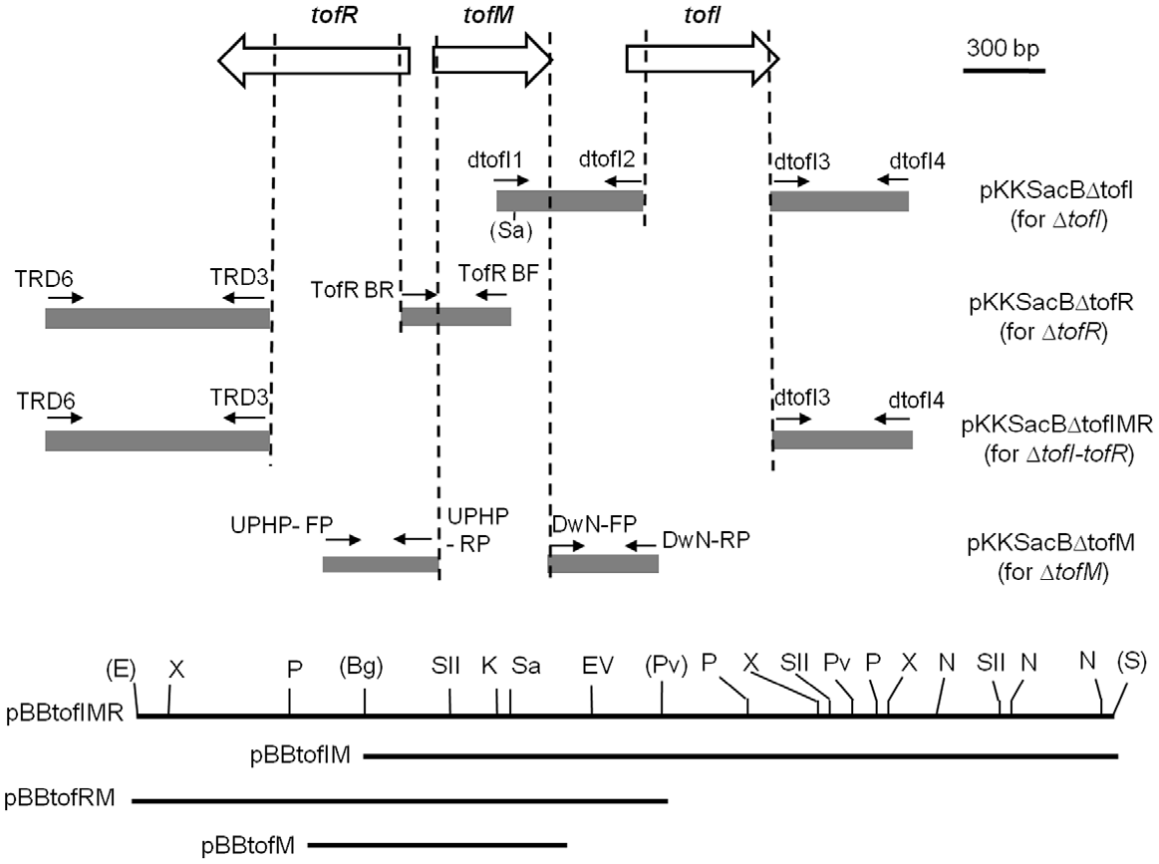
A schematic view of the *tofI*, *tofM*, and *tofR* loci and the DNA constructs used for deletion mutation and genetic complementation. The grey areas indicate the flanking regions cloned in pKKSacB for individual or combined deletions of *tofI*, *tofM*, and *tofR*. The genomic regions to be deleted with the DNA constructs in pKKSacB are indicated with vertical hatched lines, while those cloned in a broad host vector, pBBR1MCS-2 or pBBR1MCS-5, for complementation tests are indicated with horizontal solid lines. Small arrows indicate the primers (Table 2) used for the amplification of each flanking region. Abbreviation for restriction sites are as follows: Bg, *Bgl*II; E, *Eco*RI; EV, *Eco*RV; K, *Kpn*I; N, *Not*I; P, *Pst*I; Pv, *Pvu*II; S, *Sac*I; Sa, *Sal*I; SII, *Sac*II; X, *Xho*I. Restriction sites used for generating pBBtofIMR, pBBtofIM, and pBBtofRM are denoted with parentheses.

DNA constructs for deletion mutations and deletion mutants of *B. glumae* generated in this study are listed in Table 1. PCR primers used to create and confirm deletion mutations are listed in Table 2. All deletion mutants generated in this study were obtained through double-crossover homologous recombination in the flanking regions of targeted genes (Figure 1).

To construct pKKSacBΔtofI that was used to create the *tofI* deletion mutants, a 545-bp region upstream and a 512-bp region downstream of *tofI* were amplified with the primer sets, dtofI1/dtofI2 and dtofI3/dtofI4, respectively (Table 2). The resultant PCR products for these *tofI* flanking sequences were initially cloned into pSC-A-amp/kan to generate pSCtofIU and pSCtofID. The downstream region of *tofI* in pSCtofID was then sub-cloned into pLD55 [19] using *Bam*HI and *Spe*I sites to get pLDtofID. The upstream region of *tofI* in pSCtofIU was cut with *Kpn*I and *Bam*HI and was then ligated to pLDtofID, cut with the same restriction sites, to generate pLDtofIUD. Because initial attempts to generate a *tofI* deletion mutant with pLDtofIUD using the tetracycline-resistant gene in pLD55 as a counter-selection marker in the presence of fusaric acid [19] failed, the deletion construct cloned into pLD55 was moved to pKKSacB through the following steps: the 1.1-kb *Kpn*I/*Xba*I-cut fragment from pLDtofIUD was first ligated to pJP5603, cut with *Kpn*I and *Xba*I, to generate pJPtofIUD and increase the choice of restriction sites for the final cloning into pKKSacB; the 1.1-kb *Sal*I-cut fragment derived from the native *Sal*I site present 68 bp downstream from the 5′ end of the *tofI* upstream region cloned into pJPtofIUD and the *Sal*I site in the polylinker region of the same plasmid was then ligated into pKKSacB, cut with *Sal*I, to obtain pKKSacBΔtofI.

To construct pKKSacBΔtofR that was used to create the *tofR* deletion mutants, a 426-bp region upstream and an 829-bp region downstream of *tofR* were amplified with the primer sets, TofR BF/TofR BR and TRD6/TRD3, respectively (Table 2). The resultant PCR products were cloned into pSC-A-amp/kan to generate pSCtofRU and pSCtofRD, respectively. The downstream region of *tofR* in pSCtofRD was removed using the *Bam*HI site in the primer and the *Pst*I site in the polylinker region of the plasmid and subsequently ligated to pKNOCK-Km, cut with *Bam*HI and *Pst*I, to get pKKtofRD. The upstream region of *tofR* in pSCtofRU, obtained from *Spe*I and *Bam*HI digestion, was then cloned into pKKtofRD using the same restriction sites, to generate pKKtofRUD. Finally, the *Spe*I-cut 1.3-kb DNA fragment containing the recombined flanking regions of *tofR* from pKKtofRUD was cloned to pKKSacB at the *Spe*I site to obtain pKKSacBΔtofR.

To construct pKKSacBΔtofM that was used to create the *tofM* deletion mutants, a 433-bp region upstream and a 412-bp region downstream of *tofM* were amplified with the primer sets, UPHP-FP/UPHP-RP and DwN-FP/DwN-RP, respectively (Table2). The amplified PCR products were initially cloned into pSC-A-amp/kan to generate pSCtofMU and pSCtofMD, respectively (Table 2). The upstream region of *tofM*, obtained by *Bam*HI and *Spe*I digestions of pSCtofMU, was cloned into pKKSacB at the *Bam*HI and *Spe*I sites to get pKKSacBtofMU. The downstream region of *tofM*, cut from pSCtofMD by *Spe*I and *Xba*I, was then ligated to pKKSacBtofMU, cut with *Spe*I and *Xba*I, to obtain pKKSacBΔtofM.

To construct pKKSacBΔtofIMR that was used for the deletion of the entire *tofI-tofR* region, the downstream region of *tofR* in pSCtofRD was obtained by *Kpn*I and *Bam*HI digestions and subsequently ligated into pLDtofID, cut with *Kpn*I and *Bam*HI, to get pLDtofIDRD. The 1.3-kb DNA fragment that resulted from the *Spe*I digestion of pLDtofIDRD was then ligated to the *Spe*I-cut pKKSacB to obtain the final deletion construct, pKKSacBΔtofIMR.

### DNA Constructs for the Complementation of the QS Mutants

A cosmid library of the *B. glumae* 336gr-1 genome was screened with the primers, tofI-jh1 and tofI-jh2, to identify the cosmid clone that contains *tofI*. The cosmid clone, pCos808, was identified to contain *tofI* as well as *tofR* and *tofM*. pBBtofIMR, which contains *tofI*, *tofM*, and *tofR*, was constructed by cloning a 3,670-bp DNA fragment containing *tofI*, *tofM*, and *tofR* from Cos809 into pBBRMCS-2 using the *Eco*RI and *Sac*I sites. pBBtofIM was generated by subcloning the 2,808-bp *tofI/tofM* region of pBBtofIMR into pBBRMCS-5 using *Bgl*II and *Sac*I sites. pBBtofRM was constructed by subcloning the 1,925-bp *tofR/tofM* region of pBBtofIMR in pBBRMCS-2 using *EcoR*I and *Pvu*II sites. For pBBtofM, a 986-bp region that includes *tofM* was amplified using the primers orf1-CT-F and orf1-CT-R (Figure 1 and Table 2). The PCR products were initially cloned into the pSC-A-amp/kan vector following the manufacturer’s protocol to generate pSCtofM. Then, the *tofM* region of pSCtofM was subcloned into pBBR1MCS-5 using *Spe*I and *Hind*III sites to get pBBtofM.

For complementation, each of these constructs was introduced into the appropriate *B. glumae* strain through triparental mating [18].

### AHL Production Assays

*Chromobacterium violaceum* CV026, which produces the purple pigment, violacein, in the presence of AHL molecules [20], was used as a biosensor to determine the AHL production by *B. glumae*. The AHL production assay was performed following the procedure used by Kim *et* *al.* (2004) with some modifications. Briefly, the supernatant fraction of an overnight culture of each *B. glumae* strain grown in LB broth at 37°C obtained after centrifugation was extracted with an equal volume of ethyl acetate, air-dried in a fume hood, and the residue dissolved in 1% volume of sterile distilled deionized water. Then, 20 µl of each culture extract were applied to the cells of *C. violaceum* CV026 immediately after they were inoculated on a LB agar plate. The production of the purple pigment by this biosensor strain was observed after 48 h incubation at 30°C.

### Quantification of Bacterial Growth

To quantify bacterial growth in liquid and solid media, an equal amount of overnight culture per volume of medium was applied to liquid and solid media (∼10^6^ cell/ml medium). For solid medium, 12.5 µl of an overnight culture were spread on an LB agar plate containing approximately 12.5 ml of LB agar. For liquid media, 3 µl of the same overnight culture were added to 3 ml LB broth. After incubation at 37°C for 24 h, bacterial growth was determined by measuring the absorbance of the bacterial culture suspension at 600 nm (OD_600_). Overnight cultures in LB broth were measured directly. Cultures grown on LB agar plates were resuspended in 12.5 ml of fresh LB broth and then measured for OD_600_.

### Quantification of Toxoflavin Production

Toxoflavin production by each strain of *B. glumae* was quantified following a previously established method [4] with some modifications for cultures grown in both liquid and solid media. For bacteria grown in LB broth, toxoflavin present in the supernatant obtained from the centrifugation of 1 ml of culture was extracted with 1 ml of chloroform. Following centrifugation, the chloroform fraction was transferred to a new microtube and air-dried in a fume hood. The residue in the microtube was dissolved in 1 ml of 80% methanol. For bacteria grown on LB agar, bacterial cells were removed from the surface of the agar and the remaining agar containing the diffused toxoflavin was cut into small pieces with a razorblade. The chopped agar was then mixed with chloroform in 1∶1 (w/v) ratio for toxoflavin extraction and the chloroform fraction was filtered through filter paper and collected in a new microtube. Chloroform was evaporated and culture filtrate residue was dissolved in 80% methanol as previously described. The absorbance of each sample was measured at 393 nm to determine the relative amount of toxoflavin [21].

### Virulence Tests for B. Glumae

The onion assay system that was previously used to determine the virulence of *Burkholderia cenocepacia* [22] and *B. glumae* [18], [23] was adopted in this study with minor modifications. Briefly, the fleshy scales of yellow onions were cut into pieces (∼2×4 cm) with a sterile razorblade and a 2 mm-slit was made in the center of each onion piece with a sterile micropipette tip. Two microliters of bacterial suspensions made from cultures grown on a LB agar plate, suspended in 10 mM MgCl_2_ and adjusted to 5×10^7^ CFU/ml, were applied to the slit on each piece of onion scale. The inoculated onion scales were incubated in a moist chamber at 30°C for 72 h. The virulence level of each *B. glumae* strain was assessed by measuring the area of maceration on each onion scale. Virulence of *B. glumae* strains in rice was tested following a previously established method [23].

## Results

### Generation of a Series of Markerless Deletion Mutants of *tofI* and *tofR*

**Figure 2:**
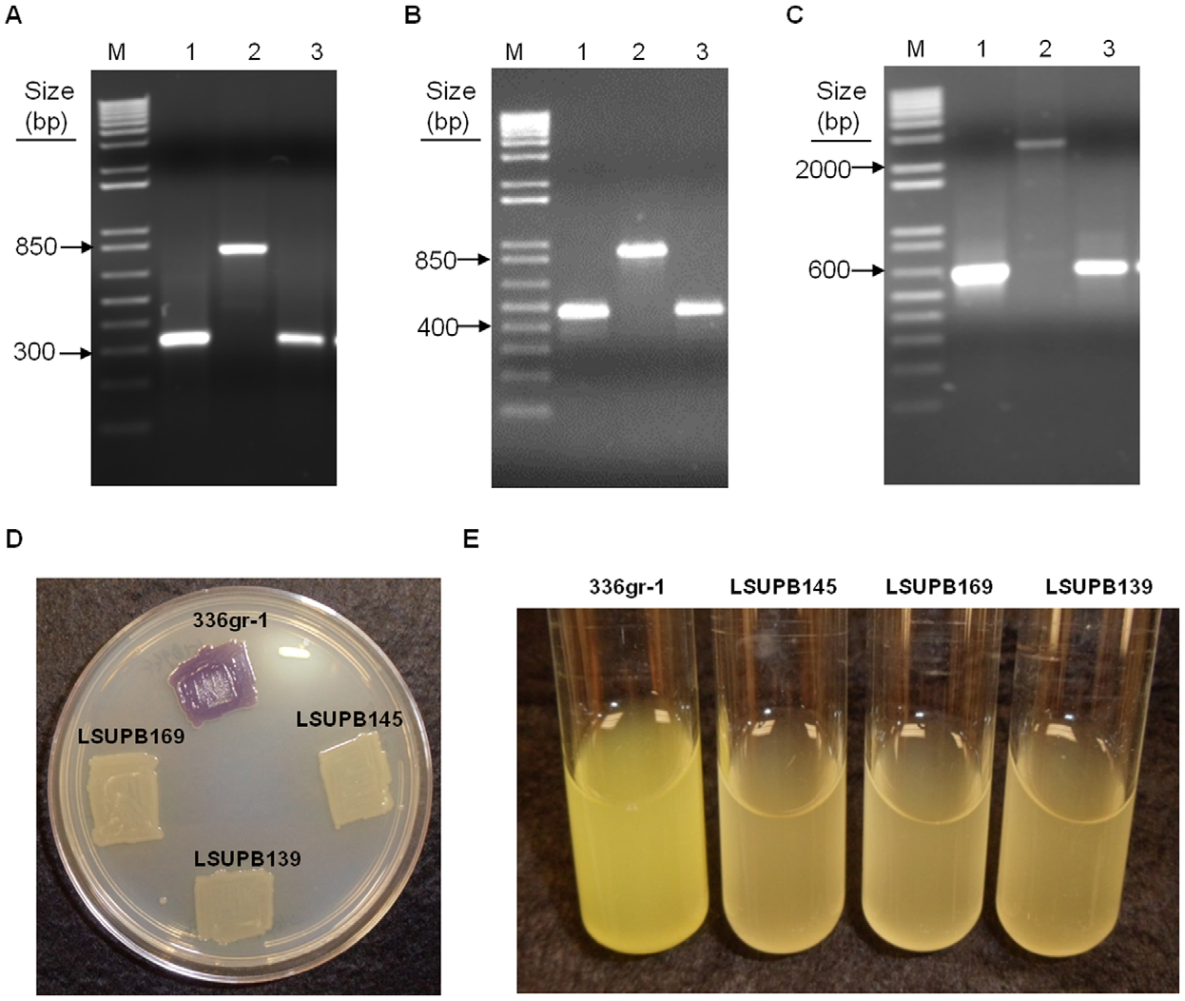
PCR products from diagnostic PCRs used to confirm deletion mutations in *Burkholderia glumae* and *N-*acyl homoserine lactone (AHL) signal production and toxoflavin production of deletion mutants. (A) PCR products amplified from primers, TofI(H)F and TofI(H)R, to confirm the *tofI* deletion in LSUPB145. Template DNA for each lane is as follows: 1, pKKSacBΔtofI; 2, genomic DNA of *B. glumae* 336gr-1; and 3, genomic DNA of *B. glumae* LSUPB145. (B) PCR products amplified with primers, TofR(H)F and TofR(H)R, to confirm the *tofR* deletion in LSUPB169. Template DNA for each lane is as follows: 1, pKKSacBΔtofR; 2, genomic DNA of *B. glumae* 336gr-1; and 3, genomic DNA of *B. glumae* LSUPB169. (C) PCR products amplified with primers, TofI(H)F and TofR(H)R, to confirm the *tofI-tofR* deletion in LSUPB139. Template DNA for each lane is as follows: 1, pKKSacBΔtofIMR; 2, genomic DNA of *B. glumae* 336gr-1; and 3, genomic DNA of *B. glumae* LSUPB139. M indicates the 1 kb Plus DNA ladder (Invitrogen, Santa Clara, CA, USA) used as a marker. (D) Violacein production, shown as a purple pigment, by the biosensor, *Chromobacterium violaceum* CV026, in the presence of the culture extracts of the *B. glumae* strains, 336gr-1, LSUPB145, LSUPB169, and LSUPB139. Photo was taken 48 h after application of bacterial culture extracts on *C. violaceum* CV026 inoculated onto a LB agar plate. (E) Toxoflavin production, shown as a yellow pigment, in the LB broth by *B. glumae* strains, 336gr-1, LSUPB145, LSUPB169, and LSUPB139. Photo was taken after 24 h incubation at 37°C.

Mutant derivatives of *B. glumae* 336gr-1 with deleted *tofI*, *tofR*, or the entire *tofI-tofR* region, including the intergenic region, were generated using the pKKSacB system (Ham and Barphagha, *unpublished*), following the procedures described in the Materials and Methods section (Table 1 and Figure 1). Genetic confirmation of the deletion mutants, LSUPB145 (*ΔtofI*), LSUPB169 (*ΔtofR*), and LSUPB139 (*ΔtofI-tofR*), was performed using PCR and primers corresponding to the DNA sequences flanking each deleted region (Figure 2 and Table 2). The size of the PCR products amplified from each mutant was the same as that of the PCR products amplified from the DNA construct used for the corresponding deletion mutation, and the size difference of the PCR products between the wild type and each mutant was matched to the predicted size of the deleted DNA sequence (Figure 2).

**Figure 3:**
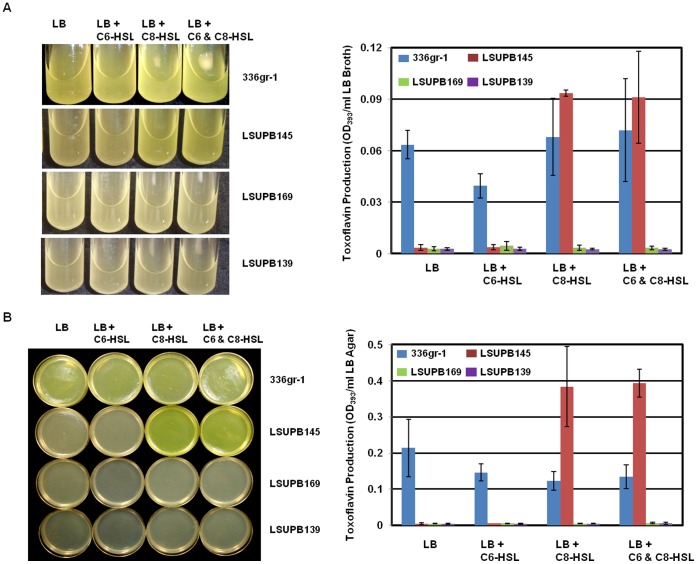
Toxoflavin production by *Burkholderia glumae* strains, 336gr-1 (wild type), LSUPB145 (*ΔtofI*), LSUPB169 (*ΔtofR*) and LSUPB139 (*ΔtofI-tofR*) in the presence or absence of 1 **µM** ***N-*** **hexanoyl homoserine lactone (C6-HSL) and** ***N-*** **octanoyl homoserine lactone (C8-HSL) in LB broth (A) and on LB agar (B).** LB broth and LB agar were inoculated with equal amounts of bacterial cells (∼10^6^ CFU) per ml of media. Bacteria were spread uniformly on LB agar plates with a spreader. Photos were taken and toxoflavin were quantified after 24 h incubation at 37°C. LB agar plates were photographed after removal of bacterial culture from the medium. Error bars indicate the standard deviation from three replications.

Mutations in *tofI* and *tofR* were also confirmed with the biosensor strain, *C. violaceum* CV026, which produces the purple pigment, violacein, in the presence of AHL compounds, including C6-HSL and C8-HSL [20]. The culture extract of the wild type strain, 336gr-1, caused the production of violacein by the biosensor, while that of the deletion mutants did not (Figure 2D), indicating that these mutants did not produce the AHL molecules required for QS. Likewise, none of the mutants produced toxoflavin in either LB agar or LB broth (Figures 2E and 3). These results were consistent with the previous study by Kim *et* *al.* (2004), which showed the dependence of toxoflavin production by *B. glumae* on *tofI* and *tofR*.

### Restoration of Bacterial Growth and Toxoflavin Production in the ***Δ*** *tofI* Strain, LSUPB145, by C8-HSL

All the QS mutants produced little toxoflavin compared to the wild type in both liquid and solid media (Figure 3). If 1 µM C8-HSL was added to the media, the *ΔtofI* mutant, LSUPB145, regained the ability to produce toxoflavin, but the *ΔtofR* mutant, LSUPB169, and the *ΔtofI-tofR* mutant, LSUPB139, did not (Figure 3). Patterns of toxoflavin production by mutant strains in the presence of exogenous synthetic AHL compounds were similar in both liquid and solid media (Figure 3). In both growth conditions, LSUPB145 appeared to produce more toxoflavin than the wild type 336gr-1 in the presence of 1 µM C8-HSL (Figure 3). According to the statistical analysis using a two-sample t-test, the toxoflavin production in 336gr-1 and LSUPB145 was significantly different from each other in solid media (T value = −3.97, P value = 0.0166) but not in liquid media (T value = −1.95, P value = 0.1888).

**Figure 4:**
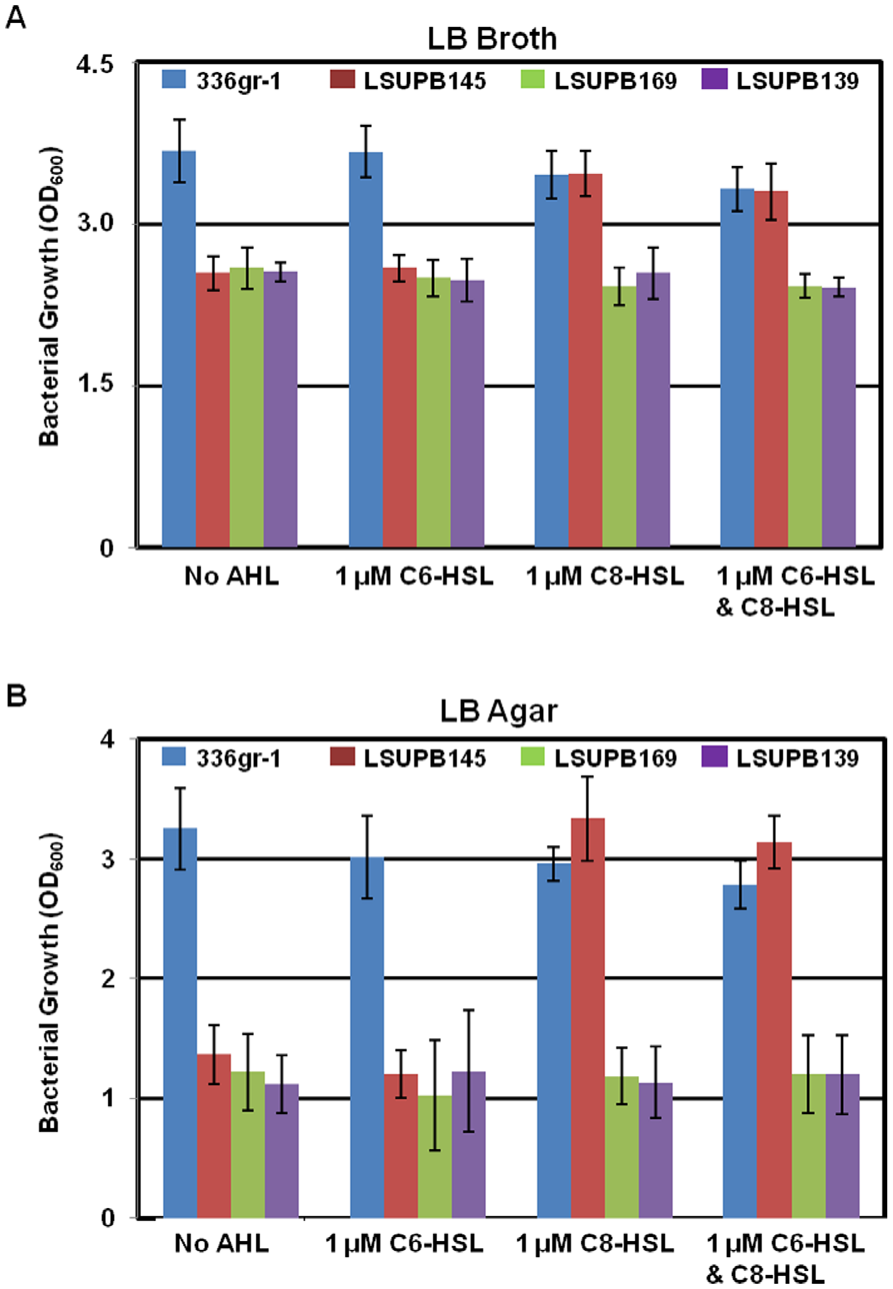
Bacterial growth of *Burkholderia glumae* strains, 336gr-1 (wild type), LSUPB145 (*ΔtofI*), LSUPB169 (*ΔtofR*) and LSUPB139 (*ΔtofI-tofR*) in the presence or absence of 1 µM *N-*hexanoyl homoserine lactone (C6-HSL) and *N-*octanoyl homoserine lactone (C8-HSL) in LB broth (A) and on LB agar (B). LB broth and LB agar were inoculated with equal amounts of bacterial cells (∼10^6^ CFU) per ml of media. Absorbance of each bacterial culture was measured after 24 h incubation at 37°C. Error bars indicate the standard deviation from three replications.

In addition, the QS mutant strains showed reduced growth when compared to the wild type in both liquid and solid media after 24 h incubation at 37°C (Figures 4 and S1). ANOVA and post hoc LSD tests validated that the observed growth reduction of all the three QS mutants in both types of medium condition was statistically significant (not shown). The difference in bacterial growth between the wild type and the QS mutants appeared to be greater in solid media than in liquid media (Figure 4). Addition of C8-HSL to both liquid and solid media restored the growth of the *ΔtofI* strain, LSUPB145, to the wild type level, but did not have any effect on the growth of the other QS mutants or the wild type strain (Figures 4 and S1). C6-HSL did not affect the growth of any strain tested (Figures 4 and S1).

### Toxoflavin Production of *ΔtofI* and *ΔtofR* Derivatives of the Wild Type Strain, 336gr-1, at High Culture Density on LB Agar

**Figure 5:**
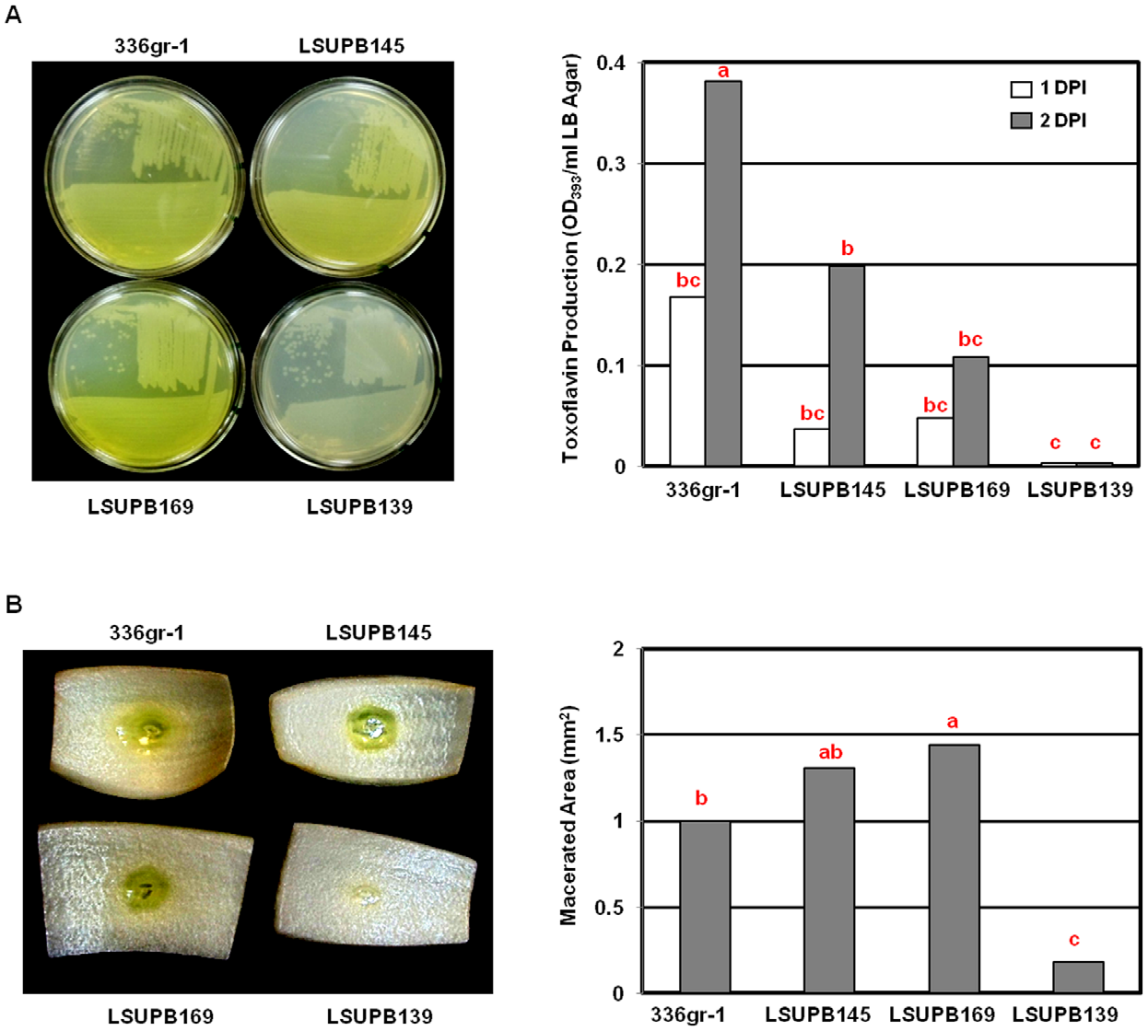
Toxoflavin production (A) and virulence phenotypes (B) by *Burkholderia glumae* strains, 336gr-1 (wild type), LSUPB145 (*ΔtofI*), LSUPB169 (*ΔtofR*) and LSUPB139 (*ΔtofI-tofR*). (A) LB agar plates inoculated with the streaking method with inoculums from fresh bacterial colonies of *B. glumae* strains. Photos were taken and quantification procedures were performed 48 h after incubation at 37°C. (B) Virulence phenotypes on onion bulb scales. Photos were taken and maceration was measured 72 h after incubation in a wet chamber at 30°C. Columns for toxoflavin production (A) and area of maceration (B) represent the mean values from three replications and five replications, respectively. The letters above columns indicate significant differences among *B. glumae* strains (*P<*0.01). DPI: days post inoculation.

*ΔtofI* and *ΔtofR* mutants, LSUPB145 and LSUPB169, respectively, produced toxoflavin when grown on solid media after inoculation with the streaking method using an inoculation loop (Figure 5A). The *ΔtofI-tofR* strain, LSUPB139, on the other hand, did not produce any detectable toxoflavin in the same condition (Fig. 5A). Even though LSUPB145 and LSUPB169 produced less amounts of toxoflavin than the wild type, 336gr-1, did in this growth condition (Figure 5A), their phenotypes were strikingly different from those shown in LB broth (Figure 3A) or LB agar inoculated with the spreading method (Figure 3B). Similar results were observed in tests with other types of solid media, including King’s B agar [24] (data not shown). In an onion assay established to indirectly determine the virulence of *B. glumae* [23], LSUPB145 and LSUPB169, but not LSUPB139, were able to cause comparable or larger maceration symptoms on onion bulb scales in comparison with the wild type (Figure 5B). Inoculums prepared from the cultures in LB broth and LB agar showed similar results (data not shown).

### Identification of a New Regulatory Gene, *tofM*, in the Intergenic Region between *tofI* and *tofR*

Based on the observation mentioned above, we speculated that toxoflavin could be produced in a *tofI-* or *tofR-*independent manner at certain growth conditions but could not be produced without both *tofI* and *tofR*. To verify this notion, a *tofI/tofR* double deletion mutant (*ΔtofI/ΔtofR*), LSUPB201, was generated through consecutive deletions of *tofI* and *tofR* and its phenotype in toxoflavin production was tested in various conditions. Unlike the *ΔtofI-tofR* strain LSUPB139, the *ΔtofI/ΔtofR* mutant LSUPB201 still produced toxoflavin on LB agar medium when it was inoculated with the streaking method (Figures S2 and 6B). The only difference between LSUPB139 (*ΔtofI-tofR*) and LSUPB201 (*ΔtofI/*Δ*tofR*) was the presence of the intergenic region between *tofI* and *tofR* (Figure 1), suggesting that unknown genetic element(s) present between *tofI* and *tofR* may be responsible for the *tofI* and *tofR-*independent production of toxoflavin. According to the annotated whole genome sequence of *B. glumae* BGR1 (NCBI Reference Sequence: NC_012721.2), the coding sequences of *tofI* (locus tag: bglu_2g14490) and *tofR* (locus tag: bglu_2g14470) are 612 bp- and 720 bp-long, respectively, and are separated by a region of DNA 799 bp in length that includes a single ORF (locus tag: bglu_2g14480) that is divergently transcribed from *tofR* (Figure 1). The deduced amino acid sequence of this ORF showed 22.4% identity to that of RsaM in *Pseudomonas fuscovaginae* [25] and was found to be highly conserved among *Burkhoderia* spp. (Table 3 and Figure S3). The DNA sequence of the *tofI-tofR* intergenic region of *B. glumae* 336gr-1 was identical to that of *B. glumae* BGR1.

**Table 3:** TofM homologs in *Burkholderia spp.* and *Pseudomonas fuscovaginae*.

Locus_tag/Gene	Protein ID (Accession #)	Organism	Identity (similarity)
bglu_2g14480	YP_002909042.1	*Burkholderia glumae* BGR1	100%
bgla_2g11060	YP_004349067.1	*B. gladioli* BSR3	80.0% (88.7%)
bgla_1p1750	YP_004362596.1	*B. gladioli* BSR3	23.7% (35.3%)
BCAM1869	YP_002234480.1	*B. cenocepacia* J2315	52.9% (59.2%)
Bcenmc03_5575	YP_001779190.1	*B. cenocepacia* MC0-3	52.2% (59.2%)
Bcen_3642	YP_623507.1	*B. cenocepacia* AU 1054	52.2% (59.2%)
Bmul_3970	YP_001583945.1	*B. multivorans* ATCC 17616	55.6% (63.4%)
Bamb_4117	YP_776004.1	*B. ambifaria* AMMD	52.9% (59.9%)
BamMC406_4582	YP_001811254.1	*B. ambifaria* MC40-6	51.6% (58.6%)
BamMC406_5824	YP_001815818.1	*B. ambifaria* MC40-6	28.0% (38.5%)
Bcep1808_5261	YP_001117675.1	*B. vietnamiensis* G4	52.2% (61.1%)
Bcep18194_B1051	YP_371809.1	*Burkholderia* sp. 383	51.6% (59.9%)
BTH_ll1511	YP_439707.1	*B. thailandensis* E264	51.3% (65.8%)
BURPS668_A1294	YP_001062291.1	*B. pseudomallei* 668	50.3% (63.1%)
BPSS0886	YP_110895.1	*B. pseudomallei* K96243	49.7% (62.4%)
BWAA1346	YP_105962.1	*B. mallei* ATCC 23344	49.7% (62.4%)
*rsaM*	CBI67624.1/RsaM	*Pseudomonas fuscovaginae* UPB0736	22.4% (37.2%)
BURPS1106A_A1576	YP_001075610.1	*B. pseudomallei* 1106a	32.5% (43.5%)
BURPS1106B_0414	ZP_04810916.1	*B. pseudomallei* 1106b	32.5% (43.5%)
BURPS668_A1657	YP_001062653.1	*B. pseudomallei* 668	32.5% (43.5%)
GBP346_B0905	EEP50658.1	*B. pseudomallei* MSHR346	32.5% (43.5%)
BPSS1179	YP_111192.1	*B. pseudomallei* K96243	28.7% (38.9%)
BURPS1710A_A0737	ZP_04955066.1	*B. pseudomallei* 1710a	17.1% (23.2%)
BURPS1710b_A0144	YP_335303.1	*B. pseudomallei* 1710b	17.1% (23.2%)
BTH_II1228	YP_439424.1	*B. thailandensis* E264	28.2% (41.2%)
Bamb_6054	YP_777932.1	*B. ambifaria* AMMD	24.2% (33.3%)
BamMC406_5825	YP_001815819.1	*B. ambifaria* MC40-6	11.5% (20.2%)

**Figure 6:**
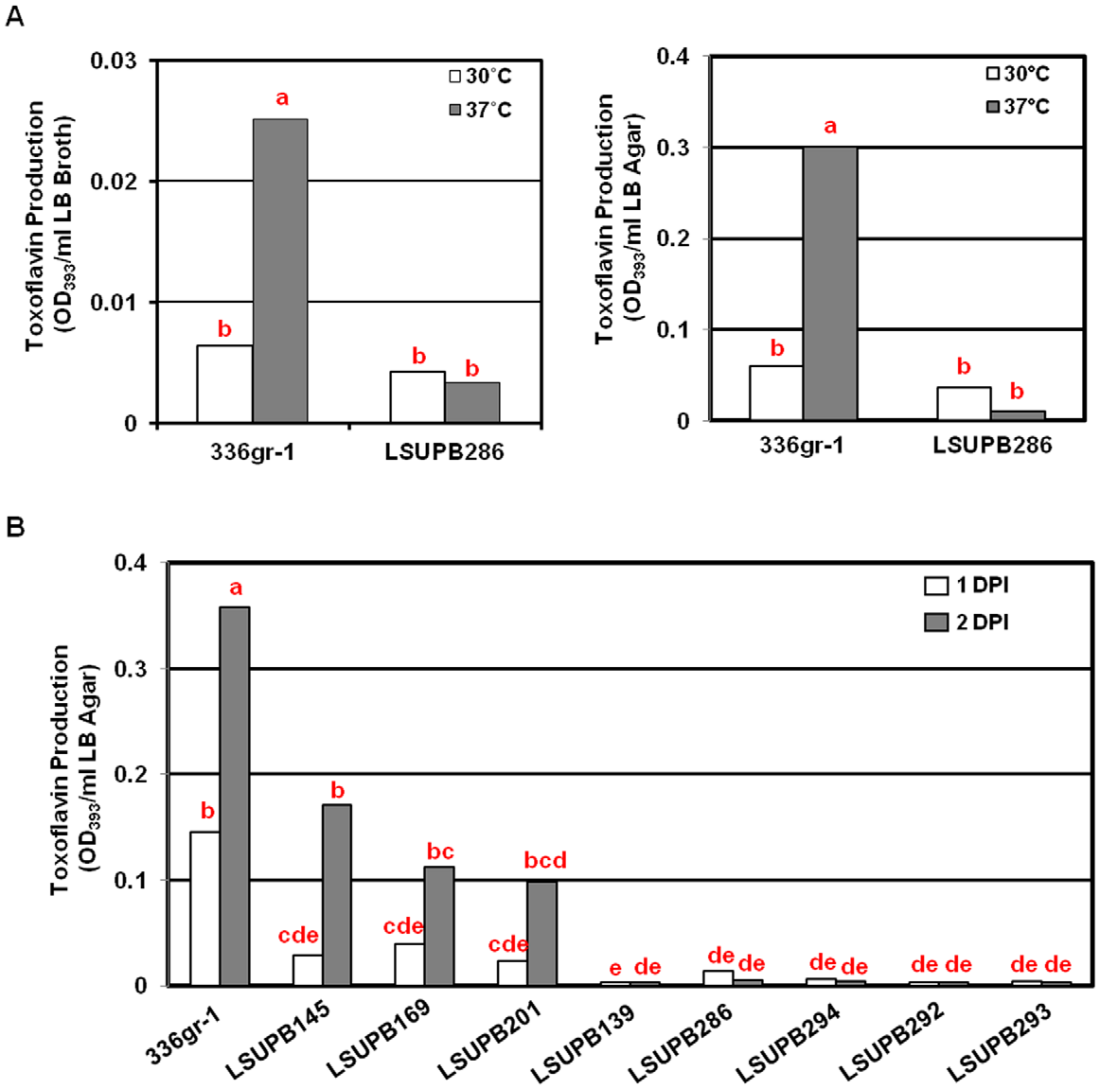
Toxoflavin production of *Burkholderia glumae tofM* deletion mutants in various genetic backgrounds. (A) Toxoflavin production by 336gr-1 (wild type) and LSUPB286 (*ΔtofM*) in LB broth (left) and on LB agar (right). Equal amounts of bacterial cells (∼10^6^ CFU/ml medium) were inoculated in both LB broth and LB agar media. For inoculation on LB agar plates, bacterial suspensions were uniformly spread with a spreader. Toxoflavin production was determined 24 h after incubation at 30°C or 37°C. (B) Toxoflavin production on LB agar plated by *B. glumae* strains, 336gr-1 (wild type), LSUPB145 (*ΔtofI*), LSUPB169 (*ΔtofR*), LSUPB201 (*ΔtofI/ΔtofR*), LSUPB139 (*ΔtofI-tofR*), LSUPB286 (*ΔtofM*), LSUPB294 (*ΔtofI/ΔtofM*), LSUPB292 (*ΔtofR/ΔtofM*) and LSUPB293 (*ΔtofI/ΔtofM/ΔtofR*). Bacteria were inoculated with the streaking method from fresh bacterial colonies. Toxoflavin production was determined 24 and 48 h after incubation at 37°C. Each column for A indicates a mean values from three replications, while that for B represents a mean value from six replications conducted in two independent experiments. The letters above columns indicate significant differences among *B. glumae* strains (*P<*0.01).

To determine the function of this ORF, deletion mutations of this ORF were made in strains with the genetic backgrounds of Δ*tofI* and Δ*tofR*, as well as the wild type background, generating LSUPB201, LSUPB292, and LSUPB286, respectively. The toxoflavin production by LSUPB286 (*ΔtofM*) was not significantly different from that by the wild type in both LB broth and LB agar conditions at 30°C (Figure 6A). However, this mutant produced a less amount of toxoflavin when compared to the wild type at 37°C and this tendency was more obvious when the bacteria were grown on LB agar medium (Figure 6A). Moreover, the same deletion in the Δ*tofI* or Δ*tofR* backgrounds resulted in an almost complete loss of the ability to produce toxoflavin, indicating that this ORF is required for the normal production of toxoflavin (Figure 6B). Thus, this ORF was considered as a functional gene and named as *tofM*, after *rsaM* due to the sequence homology and similarity in genetic location between *luxI* and *luxR* homolgs [25] (Figure S3).

Complementation with the *tofM* clone, pBBtofM, restored toxoflavin production by the Δ*tofM* strain, LSUPB286 (Figures S4B, S4C, and S5). However, complementation with this *tofM* clone did not restore the production of toxoflavin on LB agar by LSUPB294 (Δ*tofI/*Δ*tofM*), LSUPB292 (Δ*tofR/*Δ*tofM*), LSUPB293 (Δ*tofI/*Δ*tofM/*Δ*tofR*), or LSUPB139 (Δ*tofI-tofR*) (Figure S5). Complementation with pBBtofRM, which contains *tofR* and *tofM*, restored the toxoflavin-deficient phenotype of the Δ*tofR/*Δ*tofM* strain, LSUPB292, but did not restore the *tofI-*independent production of toxoflavin in LSUPB293 (Δ*tofI/*Δ*tofM/*Δ*tofR*) and LSUPB139 (Δ*tofI-tofR*) (Figure S5). Complementation with pBBtofIM, which contains *tofI* and *tofM*, did not restore the production of toxoflavin in the Δ*tofI/*Δ*tofM* mutant, LSUPB294 (Figure S5). Furthermore, complementation with pBBtofIMR, which contains *tofI*, *tofM* and *tofR*, restored the production of toxoflavin in LSUPB293 (Δ*tofI/*Δ*tofM/*Δ*tofR*), LSUPB139 (Δ*tofI-tofR*) and LSUPB201 (Δ*tofI/tofR*), but did not restore the production of toxoflavin in LSUPB286 (Δ*tofM*), LSUPB292 (Δ*tofR/*Δ*tofM*), or LSUPB294 (Δ*tofI/*Δ*tofM*) (Figures S4A and S5).

### Virulence Phenotypes of *tofI, tofR,* and *tofM* Mutants in Rice Plants

**Figure 7:**
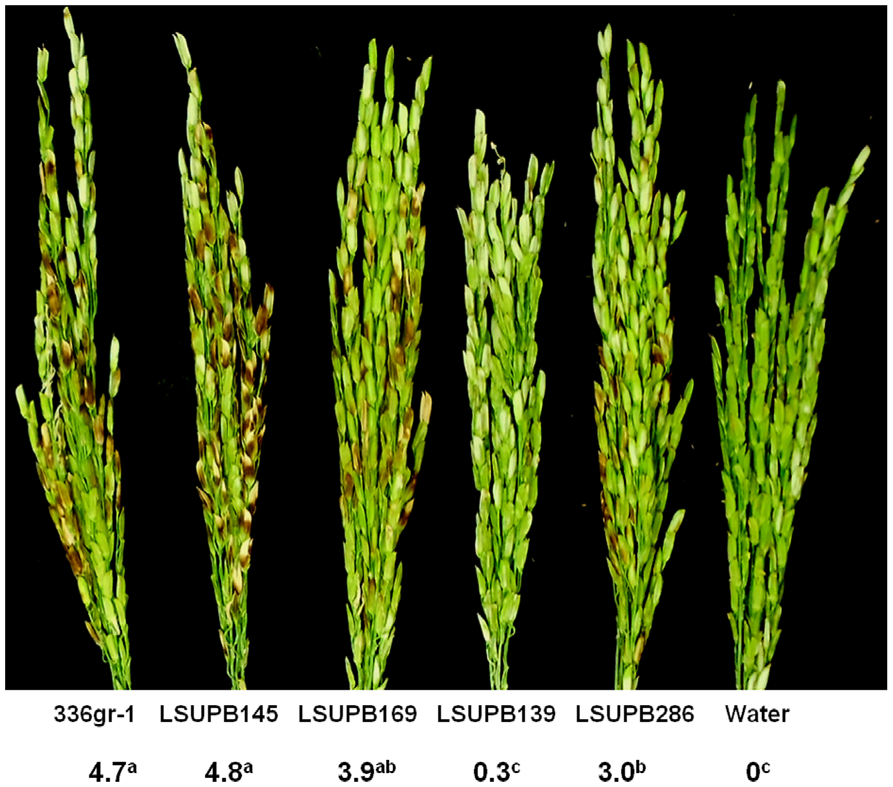
The virulence of *Burkholderia glumae* strains in rice. The numbers indicate the disease severities caused by each strain of *B. glumae* or water. Disease severity was determined with a 0–9 scale (0 - no symptom, 9– more than 80% discolored panicles) at 10 days after bacterial inoculation and each number indicates a mean value from at least five replications. The superscript letters of the disease severity values indicate significant differences among *B. glumae* strains (*P*<0.01).

In a greenhouse test, the abilities of LSUPB145 (Δ*tofI*) and LSUPB169 (Δ*tofR*) to cause symptoms in rice panicles were comparable to that of the wild type, 336gr-1 (Figure 7). However, the Δ*tofM* mutant LSUPB286 was significantly less virulent than the wild type and *tofI* or *tofR* mutants (Figure 7). In this test, LSUPB139 (Δ*tofI-tofR*) caused few visible symptoms, indicating that *tofI*, *tofR* and *tofM* are collectively required for the pathogenicity of *B. glumae* in rice (Figure 7).

## Discussion

The QS system mediated by the TofI AHL synthase and the TofR AHL receptor is known to be a central regulatory element that governs the expression of the major virulence factors of *B. glumae*, including toxoflavin [4], [8], lipase [7], and flagella [8]. In this study, a series of *tofI*, *tofM* and *tofR* mutants were generated to dissect the function of each of these QS components in the production of toxoflavin in *B. glumae*. LSUPB145 (Δ*tofI*) and LSUPB169 (Δ*tofR*) produced significantly reduced amounts of toxoflavin compared with the wild type strain, 336gr-1 (Figures 2, 3, and S4A). In addition, the ability of LSUPB145 to produce toxoflavin was restored by the addition of 1 µM C8-HSL, but not C6-HSL (Figure 3). These results were consistent with previous studies with another *B. glumae* strain, BGR1, which demonstrated the dependency of toxoflavin production on the TofI/TofR QS system and C8-HSL [4], [8]. Although TofI synthesizes both C6-HSL and C8-HSL as major products [4], the role of C6-HSL is still unknown. Notably, the *tofI* deletion mutant, LSUPB145, produced higher amounts of toxoflavin compared to the parental strain, 336gr-1, in the presence of 1 µM C8-HSL (Figure 3). This pattern was more obvious in LB agar than in LB broth (Figure 3B). This result strongly suggests that *tofI* is involved in an unknown activity that suppresses the function of C8-HSL in toxoflavin production.

Intriguingly, even though AHL signals were not produced by either the Δ*tofI* or the Δ*tofR* mutant (data not shown), both mutants were able to produce high levels of toxoflavin when inoculated with the streaking method on the LB (Figure 5) or KB agar media (data not shown). Further, LSUPB201, which has deletions of both *tofI* and *tofR*, also produced considerable amounts of toxoflavin on solid media (Figures 6 and S2). The *tofI*, *tofR* and *tofI/tofR* mutants generated via different approaches, including transposon mutagenesis and homologous recombination, produced phenotypes similar to those of the Δ*tofI*, Δ*tofR*, and Δ*tofI*/Δ*tofR* strains, indicating that the observed toxoflavin production by *tofI*, *tofR*, and *tofI/tofR* mutants is not an artifact (data not shown). Additionally, significant growth defects observed with the QS mutants suggest that the TofI/TofR QS system controls the bacterial genes required for optimal bacterial growth.

We speculated that the deviated phenotypes of LSUPB145 (*ΔtofI*) and LSUPB169 (*ΔtofR*) in toxoflavin production on solid media dependent on different methods of inoculation might be due to the differences in bacterial concentration of the initial inoculum. To test this hypothesis, an overnight culture (∼10^9^ CFU/ml) of LSUPB145 grown in LB broth was inoculated on LB agar with the streaking method, while a concentrated bacterial suspension (∼10^11^ CFU/ml) of the same strain was inoculated on LB agar with the spreading method. When an overnight culture (∼10^9^ CFU/ml) of LSUPB145 was inoculated on LB agar plates with the streaking method, the bacterial cultures frequently failed to produce toxoflavin but occasionally (with about 30% chance) produced toxoflavin (data not shown). In contrast, when a concentrated bacterial suspension (∼10^11^ CFU/ml) was inoculated on LB agar plates with the spreading method, the bacterial cultures frequently produced toxoflavin but occasionally (with about 30% chance) failed to produce toxoflavin (data not shown). In both inoculation conditions, the chance to produce toxoflavin increased as the bacterial concentration of the initial inoculum was higher (data not shown). These observations suggest that both initial concentration of bacterial inoculum and method of bacterial inoculation are critical factors for the *tofI-* or *tofR-*independent production of toxoflavin on solid media.

Based on the observed toxoflavin production by the *tofI*, *tofR* and *tofI/tofR* mutants at certain growth conditions, we speculated that *B. glumae* possesses alternative regulatory pathway(s) for the production of toxoflavin in the absence of TofI and TofR. Because the *ΔtofI-tofR* mutant, LSUPB139, did not produce toxoflavin in any growth condition tested (Figures 2, 3, 5, S2, and S4A), the intergenic region between *tofI* and *tofR* was thought to contain at least one regulatory gene that is responsible for toxoflavin production and independent of *tofI* and *tofR*. Indeed, a putative gene divergently transcribed from *tofR* was found to be involved in the production of toxoflavin and deletion of *tofM* in the wild type background caused a significant reduction in toxoflavin production and virulence in rice (Figures 6A and 7). Toxoflavin production of the Δ*tofM* strain, LSUPB286, was restored to wild type levels following complementation with the *tofM* clone, pBBtofM (Figures S4B, S4C, and S5). Nevertheless, complementation of the mutants with functional clones of the mutated genes was frequently unsuccessful (Figure S5), implying that the accurate balance of gene expression based on the correct genomic position and gene dosage of *tofI*, *tofM* and *tofR* is critical for the regulation of toxoflavin production by these genes. In this regard, it is noteworthy that the *ΔtofM* mutant was complemented by a *tofM* clone carrying *tofM* only (pBBtofM), but not by *tofM* clones carrying additional genes (pBBtofRM, pBBtofIM and pBBtofIMR); likewise, the *ΔtofRM* and *ΔtofIMR* mutants were complemented only by pBBtofRM and pBBtofIMR, respectively (Figure S5). We do not know why the *ΔtofIM* mutant could not be complemented by any clones carrying both *tofI* and *tofM*, including pBBtofIM (Figure S5).

Taken together, these results indicate that *tofM* is a positive regulator for toxoflavin production. When *B. glumae* is grown in liquid media or on solid media after inoculation with the spreading method, TofM may supplement the regulatory function of the TofI/TofR QS in the production of toxoflavin. When *B. glumae* is grown on solid media after inoculation with the streaking method, however, TofM may cause the TofI/TofR QS-independent production of toxoflavin. Even though TofM is likely a key regulatory component of the *tofI-* and *tofR*-independent pathway(s) for toxoflavin production, additional regulatory components required for the production of toxoflavin in the absence of *tofI* or *tofR* have been identified and are currently being analyzed (Chen and Ham, *unpublished*).

Even though *tofM* was identified as a positive regulator for toxoflavin production in this study, its homolog, *rsaM*, was first reported as a novel negative regulator for the QS systems of another rice pathogenic bacterium, *P. fuscovaginae* [25]. Nevertheless, *rsaM* seems to exert positive functions for virulence as well because an *rsaM* mutant of *P. fuscovaginae* showed attenuated virulence in rice [25]. Both *tofM* and *rsaM* are present in the intergenic region of *luxI* and *luxR* homologs and are oriented divergently from the *luxR* homologs (Figure S3). Recent studies on *Pseudomonas* spp. including *P. aeruginosa*, *P. putida*, and *P. fuscovaginae* revealed that *rsaL* and *rsaM*, present in the intergenic regions of *luxI* and *luxR* homologs, act as negative regulators controlling the homeostasis of AHL levels [26]. In this study, positive function of *tofM* in virulence was observed (Figure 7), however, repressive action of *tofM* on the AHL-mediated QS was somewhat ambiguous in the AHL-detection assay using the biosensor *C. violaceum* CV026 (Figure S6). The biosensor strain treated with the culture filtrate of the *tofM* mutant, LSUPB286, showed a stronger purple color than that treated with the culture filtrate of the wild type, 336gr-1 (Figure S6). However, this phenotype of LSUPB286 suggesting a negative role of *tofM* in the AHL-mediated QS could not be complemented by the *tofM* clone, pBBtofM. Quantitative analyses to precisely determine the roles of *tofM* in the expression of *tofI* and *tofR*, as well as other virulence genes, of *B. glumae* and in the production of AHL compounds are currently being conducted (Chen and Ham, *unpublished*).

A database search for *tofM* revealed that *tofM* homologs are conserved in many *Burkholderia* spp. (Table 3 and Figure S3), suggesting the importance of their functions for ecological fitness. *B. gladioli*, which also causes BPB of rice, possesses two *tofM* homologs along with two sets of *luxI* and *luxR* homologs. Between the two predicted proteins encoded by the *tofM* homologs of *B. gladioli*, one shows the highest level of homology (80% amino acid sequence identity) to TofM, while the other shows only 23.7% identity (Table 3). It is noteworthy that, among the *tofM* homologs investigated in this study, all of the homologs with greater than 49% identity in deduced amino acid sequence to *tofM* had the same position and orientation patterns as *tofM* and *rsaM* relative to their neighboring *luxI* and *luxR* homologs (Table 3 and Figure S3). Regarding the conserved genetic locations and amino acid sequences of encoded proteins, it is very probable that the *tofM* homologs of other *Burkholderia* spp., including the select agents, *B. mallei* and *B. pseudomallei*, execute similar functions to *tofM*. Thus, elucidation of the *tofM* function in the TofI/TofR QS system of *B. glumae* would provide useful insights into the counter parts of human and animal pathogenic *Burkholderia* spp.

Conclusively, *tofI-* and *tofR-*independent production of toxoflavin in *B. glumae* was revealed for the first time in this study and *tofM* was identified as a key genetic component of this newly found pathway for toxoflavin production. *tofM* alone was also found to contribute to the full virulence of *B. glumae* 336gr-1. Further studies to determine the regulatory functions of *tofM* in the expression of *tofI* and *tofR* as well as other virulence genes of *B. glumae* would lead to a better understanding of the global regulatory system that governs the expression of virulence genes in this pathogen and, possibly, other related bacterial species.

## Supporting Information

Figure S1 **Growth curves of** ***B. glumae*** **strains, 336gr-1 (wild type), LSUPB145 (** ***ΔtofI*** **), and LSUPB169 (** ***ΔtofR*** **) grown in LB broth (top left), LB broth amended with 1** **µM** ***N-*** **hexanoyl homoserine lactone (C6-HSL)(top right), and LB broth amended with or** ***N-*** **octanoyl homoserine lactone (C8-HSL)(bottom).** Bacteria were grown at 37°C in a shaking incubator at ∼200 rpm. Similar patterns of data were obtained from three independent experiments.
(TIF)

Figure S2 **Toxoflavin production by** ***B. glumae*** **strains, LSUPB145 (** ***ΔtofI*** **), LSUPB201 (** ***ΔtofI/ΔtofR*** **), LSUPB294 (** ***ΔtofI/ΔtofM*** **) and LSUPB139 (** ***ΔtofI-tofR*** **) on LB agar plates. Bacteria were inoculated on LB agar plates with the streaking method from fresh colonies of** ***B. glumae*** **strains.** Toxoflavin production is indicated by the presence of the yellow pigment in the media. Photo was taken after 24 h incubation at 37°C.
(TIF)

Figure S3 **A phylogenetic tree of the RsaM homologs found from the genome sequences of** ***Burkholderia*** **spp. and the relative positions and transcriptional directions of the** ***rsaM*** **homologs.** The accession number of TofM is indicated with a red box. Red, green, and orange arrows indicate the homologs of *luxR*, *rsaM*, and *luxI*, respectively. Arrow direction indicates the transcriptional direction of depicted genes; arrow size is not proportional to the size of the corresponding genes. The phylogenetic tree was conducted with MEGA5 [27] using the UPGMA method based on the amino acid sequences of the 27 RsaM homologs including TofM. Bootstrap values from 1000 replicates were given next to the branches. The numbers indicating the evolutionary distance at the bottom of the tree represent the number of amino acid substitutions per site.
(TIF)

Figure S4 **Toxoflavin production of** ***Burkholderia glumae*** **mutants and mutants complemented with functional clones of the mutated genes.** (A)Toxoflavin production of 336gr-1 (wild type), LSUPB145 (*ΔtofI*), LSUPB169 (*ΔtofR*), LSUPB139 (*ΔtofI-tofR*) and LSUPB139 with pBBtofIMR. (B and C) Toxoflavin production of 336gr-1 (wild type), LSUPB286 (*ΔtofM*) and LSUPB286 with pBBtofM in LB broth (B) and LB agar (C). Photos were taken at 24 h after incubation at 37°C.
(TIF)

Figure S5 **A schematic diagram summarizing the complementation tests conducted in this study.** The area deleted in each gene(s) is indicated in a lighter version of the color of the gene. *Toxoflavin production by bacteria inoculated with the streaking method.
(TIF)

Figure S6 **AHL production by** ***B. glumae*** **strains, 336gr-1 (wild type), LSUPB145 (** ***ΔtofI*** **), LSUPB286 (** ***ΔtofM*** **), and LSUPB286 complemented with pBBtofM.** AHL production by each strain of *B. glumae* is indicated by the production of violacein by the biosensor, *Chromobacterium violaceum* CV026. Photo was taken 48 h after application of *B. glumae* culture extracts on the biosensor and incubation at 30°C.
(TIF)
