# Supplementary figures and images for: Dissection of Quorum-Sensing Genes in Burkholderia glumae Reveals Non-Canonical Regulation and the New Regulatory Gene tofM for Toxoflavin Production
Source: PLoS One. 2012 Dec 20;7(12):e52150. doi: 10.1371/journal.pone.0052150 (PMC3527420; doi:10.1371/journal.pone.0052150)

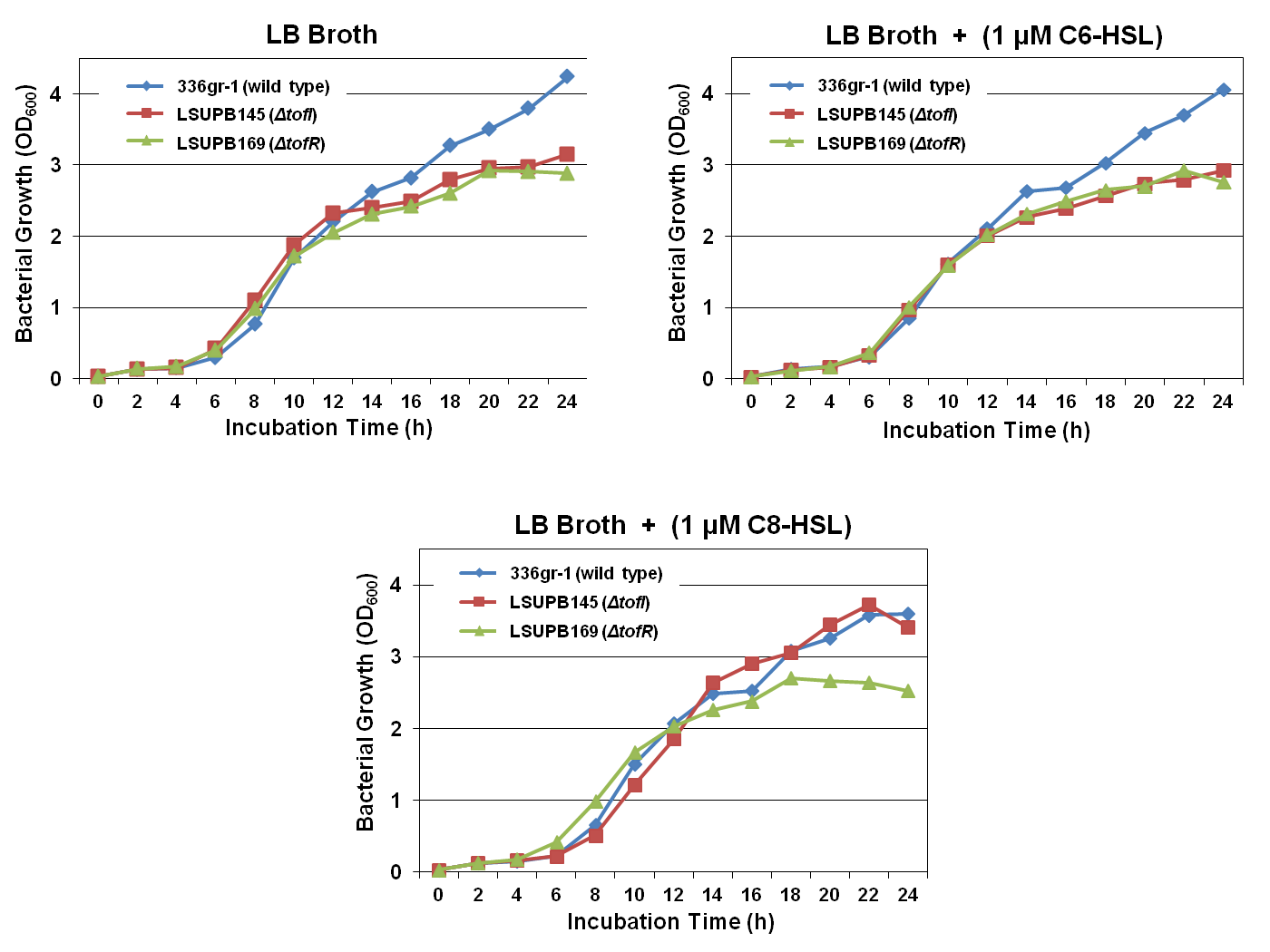

Supplement: Figure S1 — Growth curves of B. glumae strains, 336gr-1 (wild type), LSUPB145 ( ΔtofI ), and LSUPB169 ( ΔtofR ) grown in LB broth (top left), LB broth amended with 1 µM N- hexanoyl homoserine lactone (C6-HSL)(top right), and LB broth amended with or N- octanoyl homoserine lactone (C8-HSL)(bottom). Bacteria were grown at 37°C in a shaking incubator at ∼200 rpm. Similar patterns of data were obtained from three independent experiments. (TIF) [file pone.0052150.s001.tif]

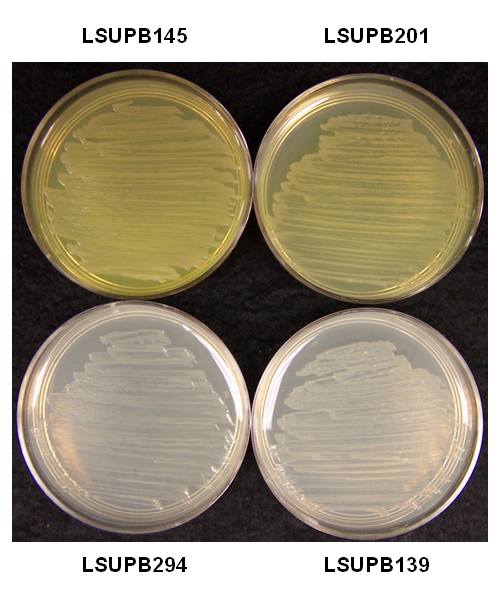

Supplement: Figure S2 — Toxoflavin production by B. glumae strains, LSUPB145 ( ΔtofI ), LSUPB201 ( ΔtofI/ΔtofR ), LSUPB294 ( ΔtofI/ΔtofM ) and LSUPB139 ( ΔtofI-tofR ) on LB agar plates. Bacteria were inoculated on LB agar plates with the streaking method from fresh colonies of B. glumae strains. Toxoflavin production is indicated by the presence of the yellow pigment in the media. Photo was taken after 24 h incubation at 37°C. (TIF) [file pone.0052150.s002.tif]

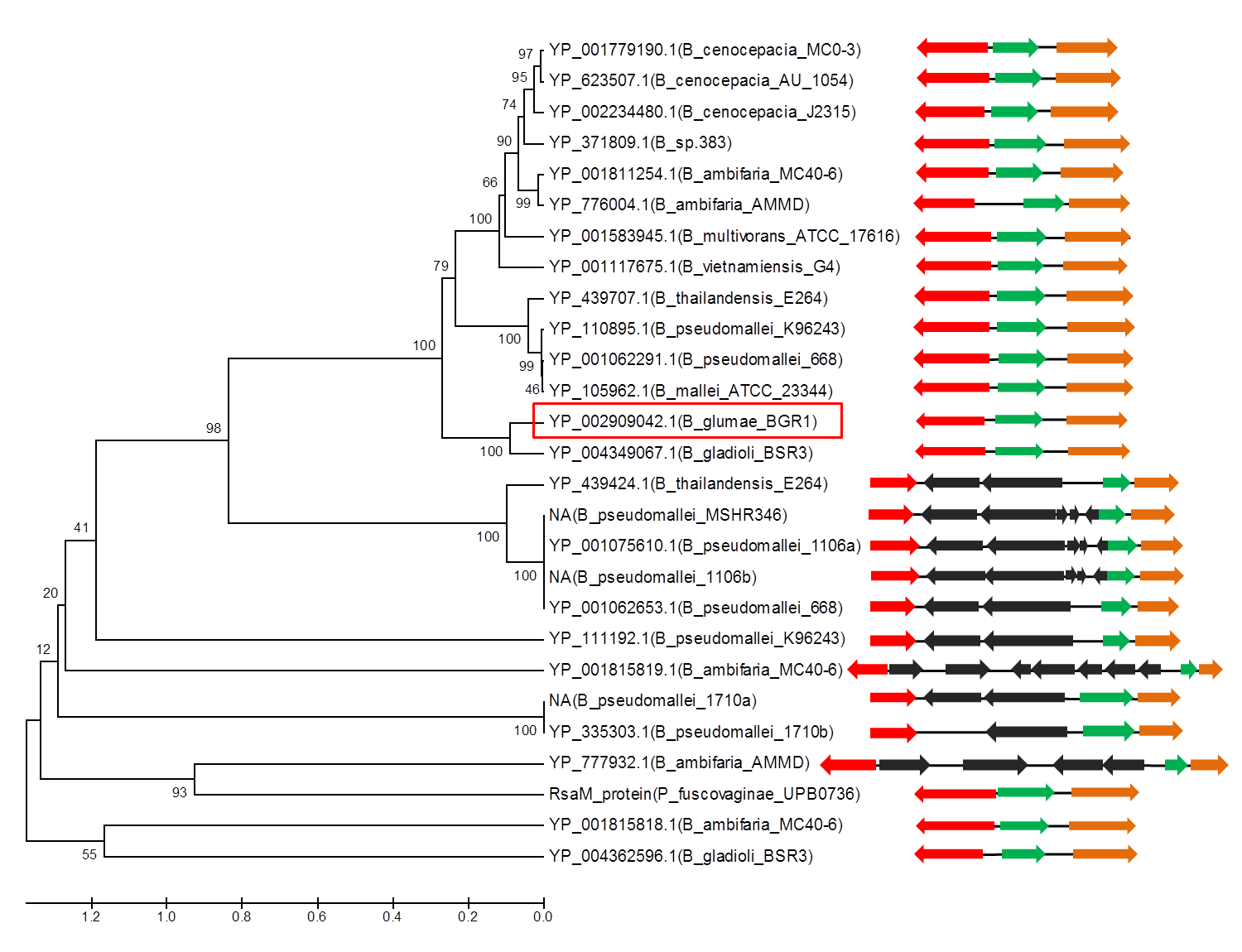

Supplement: Figure S3 — A phylogenetic tree of the RsaM homologs found from the genome sequences of Burkholderia spp. and the relative positions and transcriptional directions of the rsaM homologs. The accession number of TofM is indicated with a red box. Red, green, and orange arrows indicate the homologs of luxR, rsaM, and luxI, respectively. Arrow direction indicates the transcriptional direction of depicted genes; arrow size is not proportional to the size of the corresponding genes. The phylogenetic tree was conducted with MEGA5 [27] using the UPGMA method based on the amino acid sequences of the 27 RsaM homologs including TofM. Bootstrap values from 1000 replicates were given next to the branches. The numbers indicating the evolutionary distance at the bottom of the tree represent the number of amino acid substitutions per site. (TIF) [file pone.0052150.s003.tif]

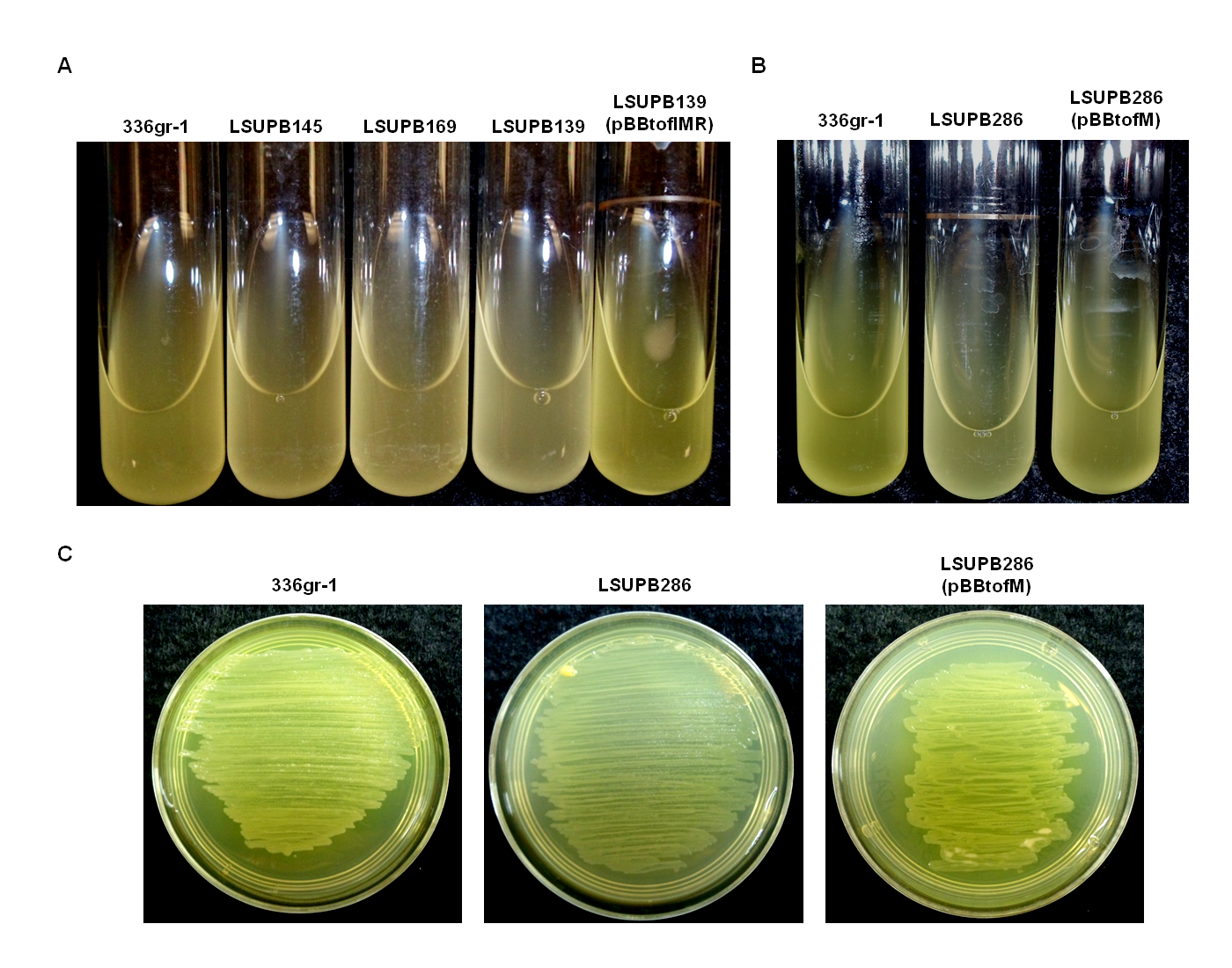

Supplement: Figure S4 — Toxoflavin production of Burkholderia glumae mutants and mutants complemented with functional clones of the mutated genes. (A)Toxoflavin production of 336gr-1 (wild type), LSUPB145 (ΔtofI), LSUPB169 (ΔtofR), LSUPB139 (ΔtofI-tofR) and LSUPB139 with pBBtofIMR. (B and C) Toxoflavin production of 336gr-1 (wild type), LSUPB286 (ΔtofM) and LSUPB286 with pBBtofM in LB broth (B) and LB agar (C). Photos were taken at 24 h after incubation at 37°C. (TIF) [file pone.0052150.s004.tif]

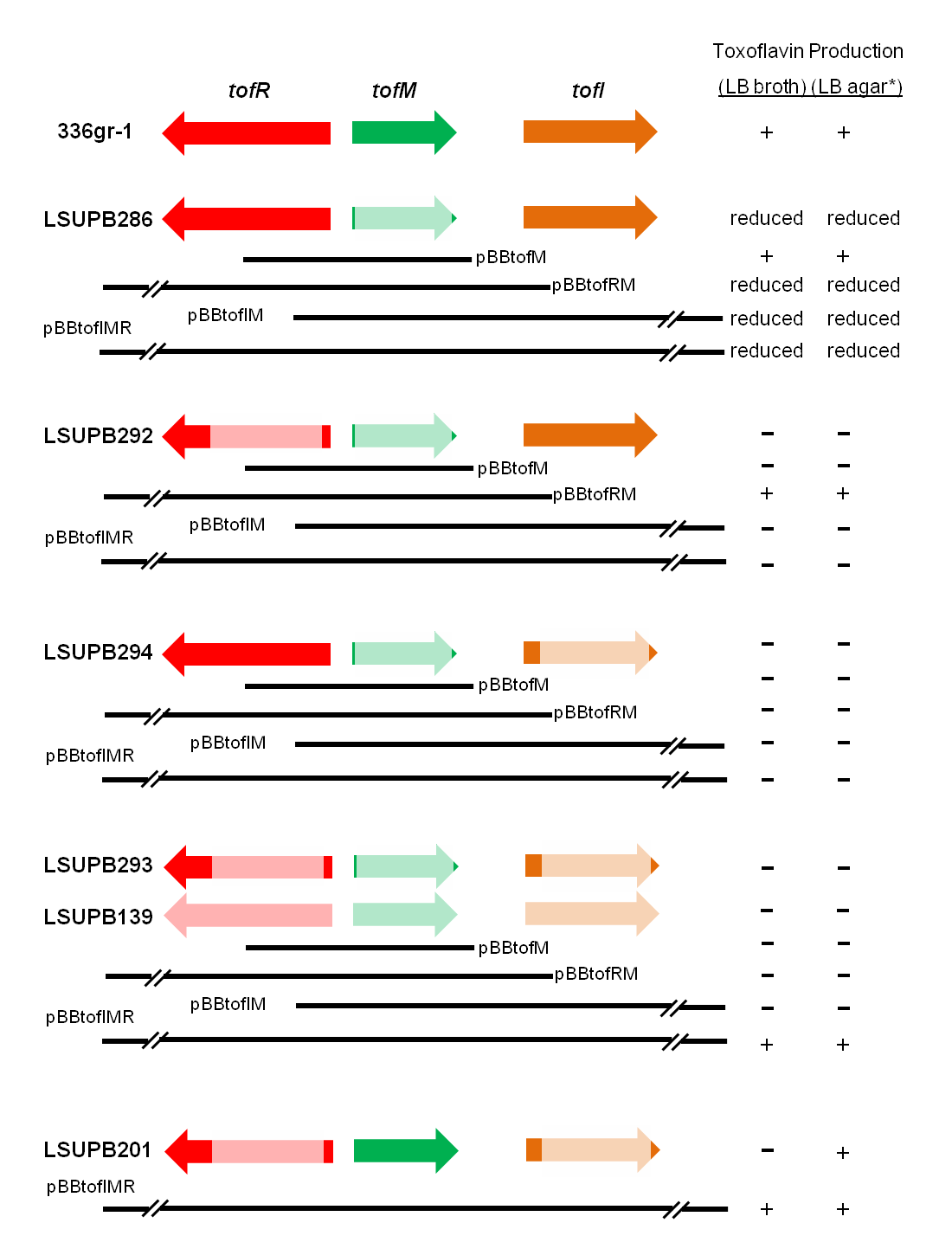

Supplement: Figure S5 — A schematic diagram summarizing the complementation tests conducted in this study. The area deleted in each gene(s) is indicated in a lighter version of the color of the gene. *Toxoflavin production by bacteria inoculated with the streaking method. (TIF) [file pone.0052150.s005.tif]

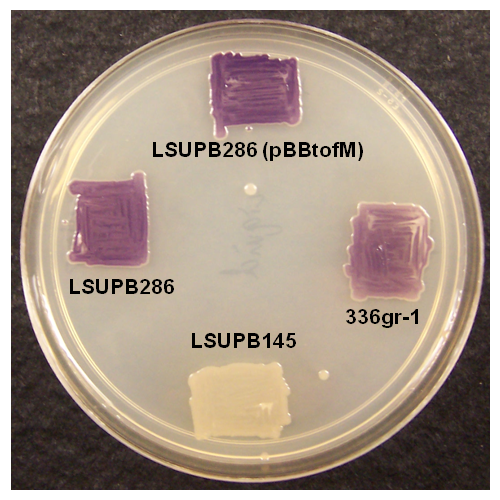

Supplement: Figure S6 — AHL production by B. glumae strains, 336gr-1 (wild type), LSUPB145 ( ΔtofI ), LSUPB286 ( ΔtofM ), and LSUPB286 complemented with pBBtofM. AHL production by each strain of B. glumae is indicated by the production of violacein by the biosensor, Chromobacterium violaceum CV026. Photo was taken 48 h after application of B. glumae culture extracts on the biosensor and incubation at 30°C. (TIF) [file pone.0052150.s006.tif]
